# Supplementary material for: In vivo intraoral waterflow quantification reveals hidden mechanisms of suction feeding in fish
Source: eLife. 2022 Feb 22;11:e73621. doi: 10.7554/eLife.73621 (PMC8906803; doi:10.7554/eLife.73621)
Supplement: Figure 2—source data 1. [file elife-73621-fig2-data1.docx]

**Figure 2—source data 1. Normalized displacement components along the three cranium-bound orthogonal axes shown in Figure 2 (mean ± SD in %) for the food and water tracers in carp and tilapia.**

|  | **Carp** | | **Tilapia** | |
| --- | --- | --- | --- | --- |
|  | **food** | **water** | **food** | **water** |
| **Intake** |  |  |  |  |
| Anteroposterior (x) | 64 ± 1 | 64 ± 1 | 57 ± 1 | 63 ± 0.1 |
| Dorsoventral (y) | 26 ± 1 | 28 ± 1 | 20 ± 0.4 | 14 ± 0.3 |
| Lateromedial (z) | 10 ± 1 | 8 ± 0.4 | 23 ± 0.1 | 22 ± 0.1 |
| **Reverse flow** |  |  |  |  |
| Anteroposterior (x) | 52 ± 1 | 54 ± 1 | 39 ± 1 | 46 ± 3 |
| Dorsoventral (y) | 29 ± 1 | 30 ± 1 | 19 ± 1 | 20 ± 1 |
| Lateromedial (z) | 19 ± 1 | 16 ± 1 | 42 ± 1 | 34 ± 2 |
| **Backward flow** |  |  |  |  |
| Anteroposterior (x) | 53 ± 1 | 58 ± 1 | 43 ± 1 | 51 ± 2 |
| Dorsoventral (y) | 29 ± 0.5 | 27 ± 1 | 23 ± 1 | 22 ± 1 |
| Lateromedial (z) | 18 ± 1 | 15 ±1 | 34 ± 1 | 27 ± 2 |
